# Fabrication of Mouse Embryonic Stem Cell-Derived Layered Cardiac Cell Sheets Using a Bioreactor Culture System

**DOI:** 10.1371/journal.pone.0052176

**Published:** 2012-12-20

**Authors:** Katsuhisa Matsuura, Masanori Wada, Kanako Konishi, Michi Sato, Ushio Iwamoto, Yuko Sato, Aki Tachibana, Tetsutaro Kikuchi, Takahiro Iwamiya, Tatsuya Shimizu, Jun K. Yamashita, Masayuki Yamato, Nobuhisa Hagiwara, Teruo Okano

**Affiliations:** 1 Institute of Advanced Biomedical Engineering and Science, Tokyo Women’s Medical University, Tokyo, Japan; 2 Department of Cardiology, Tokyo Women’s Medical University, Tokyo, Japan; 3 ABLE Corporation, Tokyo, Japan; 4 Asahi Kasei Co. LTD., Tokyo, Japan; 5 CellSeed Inc., Tokyo, Japan; 6 Laboratory of Stem Cell Differentiation, Stem Cell Research Center, Institute for Frontier Medical Sciences, Kyoto University, Kyoto, Japan; Centro Cardiologico Monzino, Italy

## Abstract

Bioengineered functional cardiac tissue is expected to contribute to the repair of injured heart tissue. We previously developed cardiac cell sheets using mouse embryonic stem (mES) cell-derived cardiomyocytes, a system to generate an appropriate number of cardiomyocytes derived from ES cells and the underlying mechanisms remain elusive. In the present study, we established a cultivation system with suitable conditions for expansion and cardiac differentiation of mES cells by embryoid body formation using a three-dimensional bioreactor. Daily conventional medium exchanges failed to prevent lactate accumulation and pH decreases in the medium, which led to insufficient cell expansion and cardiac differentiation. Conversely, a continuous perfusion system maintained the lactate concentration and pH stability as well as increased the cell number by up to 300-fold of the seeding cell number and promoted cardiac differentiation after 10 days of differentiation. After a further 8 days of cultivation together with a purification step, around 1×10^8^ cardiomyocytes were collected in a 1-L bioreactor culture, and additional treatment with noggin and granulocyte colony stimulating factor increased the number of cardiomyocytes to around 5.5×10^8^. Co-culture of mES cell-derived cardiomyocytes with an appropriate number of primary cultured fibroblasts on temperature-responsive culture dishes enabled the formation of cardiac cell sheets and created layered-dense cardiac tissue. These findings suggest that this bioreactor system with appropriate medium might be capable of preparing cardiomyocytes for cell sheet-based cardiac tissue.

## Introduction

Within the rapidly advancing field of regenerative medicine, cell-based therapies have emerged as a promising alternative to cardiac transplantation for the management of damaged heart tissue. We previously developed an original scaffold-free tissue engineering technology, designated “cell sheet-based tissue engineering”, using temperature-responsive culture dishes covalently bound to the temperature-responsive polymer poly(*N*-isopropylacrylamide) [1]. Lowering the culture temperature promotes rapid transition of the surface from hydrophobic to hydrophilic, which enables us to collect a viable monolayer cell sheet with full preservation of cell–cell contacts and extracellular matrices [2]. Many studies have reported that cell sheet-based transplantation using various types of cells improves the cardiac function of injured hearts [3], [4], [5], [6], [7]. However, recent evidence suggests that the paracrine effects, angiogenesis and cardioprotection mediated by secreted factors derived from the transplanted cells might be the major mechanisms underlying the cell transplantation-mediated improvement in cardiac function [6], [7]. Indeed, these effects might be a prerequisite to creating transplantable bioengineered thickened cardiac tissue that directly contributes to contraction.

Methods to create bioengineered cardiac tissue grafts have been established for decades [8] and we previously reported that the repeated transplantation of triple layered neonatal rat cardiac grafts onto subcutaneous tissue in vivo enables creation of cardiac tissue of about 1 mm thickness [9]. However, mammalian cardiomyocytes lose their proliferative ability soon after birth [10]; thus, pluripotent stem cells such as embryonic stem (ES) cells and induced pluripotent stem (iPS) cells are promising cell sources of cardiomyocytes. Various methods have been developed for the expansion and cardiac differentiation of ES and iPS cells [11], [12], [13], [14], [15], and embryoid body (EB) suspension culture has been widely used as an easily scalable method. Zandstra et al reported for the first time the scalable culture of mouse ES (mES) cells for cardiomyocyte production using spinner flasks [11], and Wang et al reported mES culture for cardiac differentiation using a rotary cell culture system [15]. We reported that mES cells proliferate and differentiate into cardiomyocytes in a spinner flask and that cardiac cell sheets derived from mES cells have an electrophysiological function that propagates over the cell sheets [16]. Nevertheless, when mES cells are cultured in a spinner flask it is difficult to stably maintain the cell culture condition as cells proliferate, which might affect cell growth, differentiation and viability. Recent reports have suggested that bioreactor systems with oxygen control [12] and automated fill-and-draw medium change [13] enhance cardiomyocyte production. However, it remains unclear whether cardiomyocytes from bioreactor systems are suitable for creating cardiac cell sheets.

In the present study, we created the layered cardiac cell sheets using a three-dimensional bioreactor-based suspension culture system for expanding mES cells and cardiomyocytes.

## Results

**Figure 1:**
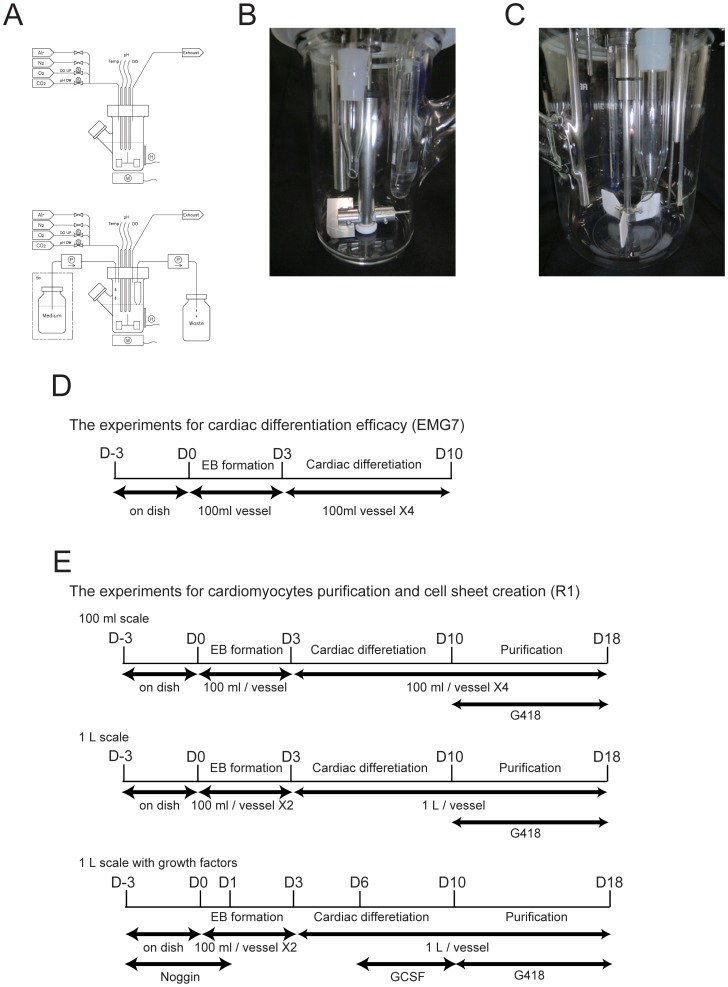
Bioreactor system and culture process. (A–C) Bioreactor system. (A) Schematic of the bioreactor system with intermittent (upper) and continuous (lower) medium exchange systems. (B) A photograph of the 100-ml bioreactor. (C) A photograph of the 1-L bioreactor. (D, E) Cell culture process in the bioreactor. (D) Schematic of the culture conditions in the bioreactor system for the evaluation of cardiac differentiation efficacy. (E) Schematic of the culture conditions in the bioreactor system for cardiomyocyte purification. Upper, 100-ml culture. Middle, 1−L culture. Lower, 1−L culture with growth factors.

**Figure 2:**
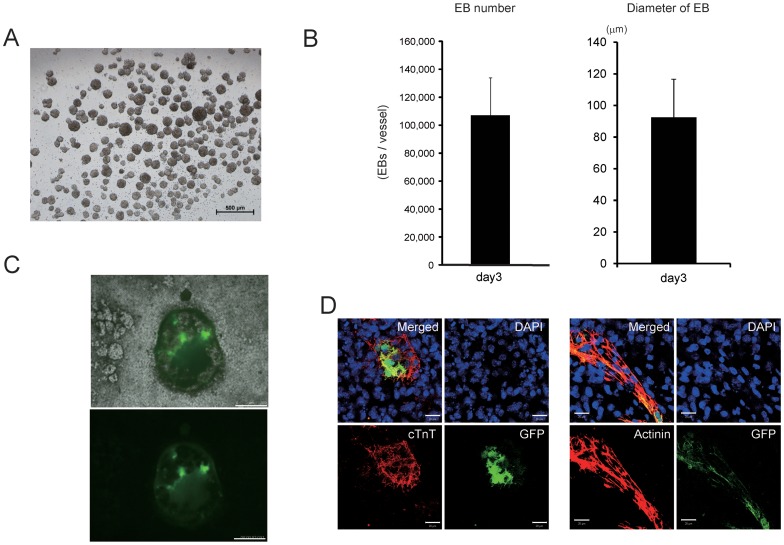
Stirred suspension culture in the bioreactor system creates a large number of EBs. (A) Representative image of EBs at 3 days of culture in the bioreactor system. Original magnification, ×10. (B) The number and diameter of EBs in the culture vessel at 3 days. (C, D) The cardiac differentiation potential of EBs created using the bioreactor system. (C) At 7 days after adherent culture of EBs created using the bioreactor system, some EBs expressed GFP. The upper panel is a merged image of phase contrast and fluorescence images. The lower panel shows GFP expression. Scale bars, 500 µm. (D) GFP(+) cells in EBs expressed several cardiac contractive proteins (red) including cardiac troponin T (cTnT, left) and sarcomeric α-actinin (right) in a fine striated pattern. Nuclei were counterstained with DAPI (blue). Scale bars, 20 µm.

Because embryoid body (EB) formation has been successfully used to promote cardiomyocyte derivation from ES/iPS cells, we attempted to create EBs in a three-dimensional suspension bioreactor culture system (Figure 1A, B). A single cell suspension of mES cells (EMG7) expressing GFP under the control of the α-myosin heavy chain (αMHC) promoter were seeded into the culture vessel of the bioreactor system, and cells were cultured without leukemia inhibitory factor and stirred. After 3 days of culture in the bioreactor system, EBs were inoculated into the turbulent flow ( Video S1) and more than 1×10^5^ EBs per vessel were created (Figure 2A, B). The mean diameter of EBs was 92 µm (92±23 µm, Figure 2B). When EBs were seeded onto gelatin-coated dishes, their spontaneous beating was observed (Video S2) at 7 days in adherent culture, and the beating cells also expressed GFP (Figure 2C). Furthermore, these GFP(+) cells also expressed some cardiac contractive proteins including α-actinin and cardiac troponin T in a fine striated pattern (Figure 2D), suggesting that EBs created with the bioreactor system might be appropriate for creating cardiomyocytes.

**Figure 3:**
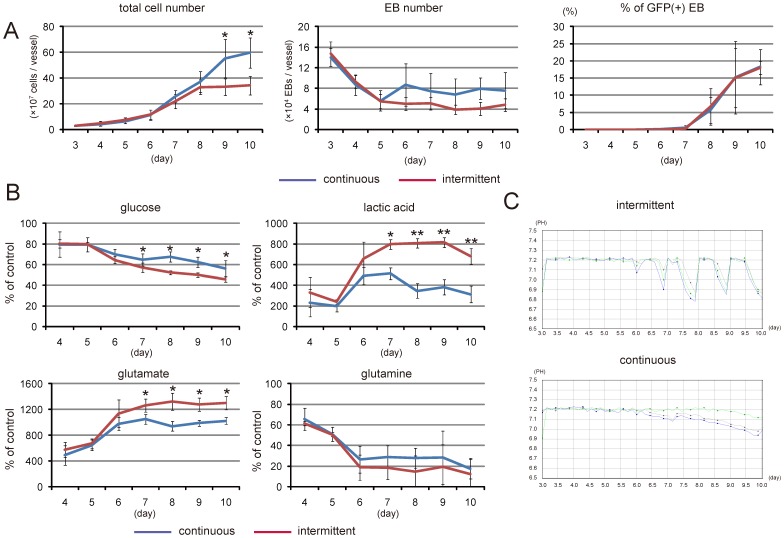
Expansion of ES cells in the bioreactor system. (A) Total cell number (left), the number of EBs (middle) in a vessel, and the percentage of GFP(+) EBs (right). Blue lines indicate the results of continuous medium exchange (*n* = 6), and red lines indicate the results of intermittent medium exchange (*n* = 6). *p<0.05. (B) Biochemical analysis of culture supernatants during bioreactor culture. Blue lines indicate the results of continuous medium exchange (*n* = 6), and red lines indicate the results of intermittent medium exchange (*n* = 6). *p<0.05, **p<0.01. (C) Trend graphs of the pH in bioreactor cultures. Upper, intermittent medium exchange; lower, continuous medium exchange. The Y-axis indicates pH, and the X-axis indicates the day of culture. Each line in the graphs indicates the pH trend of three independent experiments.

**Figure 4:**
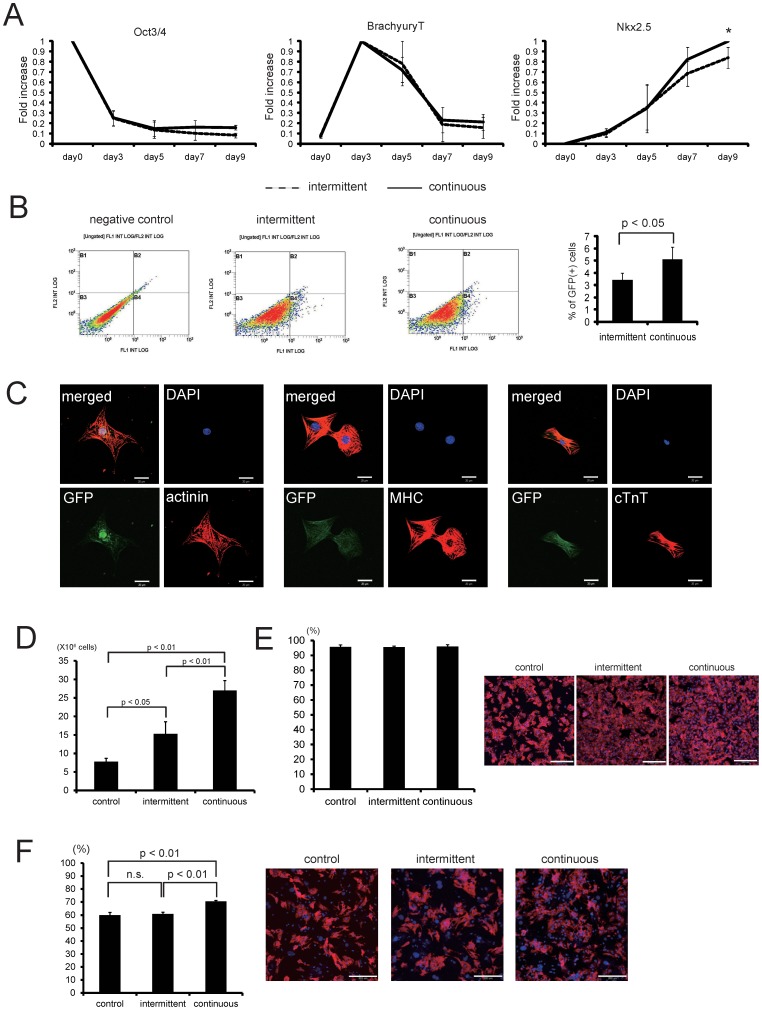
Cardiac differentiation of ES cells in the bioreactor. (A) Real-time PCR analysis (*n* = 3). Solid lines indicate the results of continuous medium exchange, and dotted lines indicate the results of intermittent medium exchange. *p<0.05. (B) Representative dot plots of flow cytometric analyses of GFP(+) cells in EBs at day 10. The graph shows the percentage of GFP(+) cells at day 10 (*n* = 6). (C) GFP(+) cells were collected using a fluorescence-activated cell sorter and then seeded onto gelatin-coated dishes. GFP(+) cells expressed sarcomeric α-actinin (upper), myosin heavy chain (MHC, middle) and cardiac troponin T (lower) in a fine striated pattern. Nuclei were counterstained with DAPI. Scale bars, 20 µm. (D, E) Comparison of the numbers of cardiomyocytes among culture conditions using R1 ES cells. (D) The number of remaining cells 8 days after starting culture with G418 (*n* = 3). Data are means ± s.d. (E) At 8 days after starting culture with G418 in the bioreactor with or without regulation, the cells were dissociated and seeded onto 1% gelatin-coated 24-well plates. At 24 h after seeding, the cells were fixed and immunostained for sarcomeric-α actinin. The percentage of sarcomeric α-actinin-positive cells among the remaining cells was calculated and is shown in the graph (*n* = 3). Data are means ± s.d. Right, representative images. Nuclei were stained with DAPI. Bars, 200 µm. (F) Comparison of cardiomyocyte engraftment between medium exchange systems. Purified cardiomyocytes after treatment with G418 were co-cultured with fibroblasts at the ratio of 8∶2. At day 2, cells were fixed and immunostained with cTnT. The percentage of cTnT-positive cells was calculated and is shown in the graph (*n* = 3). Data are means ± s.d. n.s., not significant. Right, representative images. Nuclei were stained with Hoechst33258. Bars, 200 µm.

Next we attempted to expand cells after EB formation and to induce cardiac differentiation in a three-dimensional suspension culture using the bioreactor system. After 3 days in culture, EBs in the culture vessel were collected and re-seeded into four new vessels under the same culture conditions (Figure 1D). The culture medium was then completely changed every day. To examine suitable conditions for further cultivation in terms of expansion and cardiac differentiation, dissolved oxygen conditions and agitation rate were evaluated. As shown in Figures S1 and S2, 40% saturation and 85 rpm were the most effective conditions for promoting cell proliferation and cardiac differentiation, and these conditions were used in the following experiments. Daily measurements revealed that the cell number increased in a time-dependent manner until day 8, and thereafter the cell number remained at 3.5×10^8^ (3.5±0.1×10^8^ cells per vessel) (Figure 3A, intermittent). However, the number of EBs did not change significantly from day 4 to 10 (Figure 3A). When we examined cardiac differentiation based on GFP expression in EBs, about 18% of EBs expressed GFP (Figure 3A) and 3.4% of all cells were still positive for GFP on day 10 (Figure 4B).

The cardiac differentiation of mES cells in the bioreactor system was also confirmed by quantitative RT-PCR (Figure 4A). The mRNA for the pluripotency marker Oct3/4 showed time-dependent downregulation, while that for Brachyury T, a mesodermal transcription factor, was upregulated until day 3, and that for Nkx2.5, a cardiac-specific transcription factor, was upregulated until day 9 (Figure 4A). When GFP(+) cells were isolated using a fluorescence-activated cell sorter, GFP(+) cells also expressed several cardiac contractive proteins including the sarcomeric α-actinin, myosin heavy chain (MHC) and cardiac troponin T in a fine striated pattern (Figure 4C). These findings indicate that three-dimensional stirred suspension culture in the bioreactor system induced cardiac differentiation.

During expansion and cardiac differentiation in the bioreactor culture system, the consumption of glucose and glutamine was significantly increased, and the production of lactate and glutamate was also increased (Figure 3B). Consistent with lactate accumulation, the pH of the medium also decreased during the latter part of the day from day 6 (Figure 3C). As we observed that the cells grew slowly from day 8, we speculated that the decrease in the level of glucose and the lactate-mediated decrease in pH might have hindered cell proliferation.

Because bioreactor cultures with intermittent medium exchanges could not stably maintain the medium over the culture period, we examined the effects of a continuous medium exchange system on cell proliferation and cardiac differentiation (Figure 1A, right). As shown in Figure 3B, the continuous medium exchange system significantly attenuated the decrease in glucose and glutamine concentrations in the culture medium. It also inhibited the accumulation of lactate and glutamate seen with the intermittent medium exchange condition, which involved a manual daily change of the medium. Consistent with the decrease in lactate concentration, extremely low pH was also prevented (Figure 3C). Furthermore, we observed a significant increase in the total cell number from day 9 compared with that under the intermittent medium change condition (Figure 3A). Although the percentage of GFP(+) EBs was identical between the two conditions (Figure 3A), the percentage of GFP(+) cells was significantly increased in the continuous medium exchange system, compared with that in the intermittent medium exchange system at day 10 (Figure 4B). Moreover, the levels of mRNAs for cardiac genes including Flk-1, Nkx2.5, GATA4, myosin light chain (MLC)-2a and MLC-2v at day 9 were significantly higher in the continuous medium exchange system than in the intermittent medium exchange system (Figure 4A, Figure S3). Furthermore, when R1 mES cells expressing a neomycin-resistant gene under the control of the αMHC promoter [16] (Figure 1E) were cultured in the bioreactor system, the number of cardiomyocytes in the continuous medium exchange system was significantly greater than that in the intermittent medium exchange system after G418 selection (Figure 4D, E). Conversely, without regulation, when cells were cultured in a similar bioreactor vessel in the CO2 incubator, the number of cardiomyocytes was decreased (control, Figure 4D, E). These findings strongly indicate that maintaining the culture under an appropriate condition might promote the proliferation and cardiac differentiation of mES cells, which enabled us to generate a higher number of cardiomyocytes.

Although we previously reported that cardiac cell sheets were created when mES-derived cardiomyocytes purified with G418 were co-cultured with fibroblasts at a ratio of 8∶2 on temperature-responsive culture dishes [16], the percentage of cardiomyocytes forming cell sheets was approximately 60% (unpublished data). Because it would be important to increase the survival of cardiomyocytes in cell sheets to create a more functional tissue, we examined the effects of differences in culture system on the engraftment of cardiomyocytes in cell sheets in vitro. When cardiomyocytes from the bioreactor with intermittent medium exchange were co-cultured with fibroblasts, the percentage of surviving cardiomyocytes was 61% (61.0±1.2), identical to the results obtained with cardiomyocytes cultured in the bioreactor without regulation (control, Figure 4F). However, cardiomyocytes from the bioreactor with continuous medium exchange showed a remarkable increase in survival up to 70.5% (70.5±0.7, Figure 4F). These findings suggest that a better culture environment in the process of cardiac differentiation might enhance cardiomyocyte survival in cell sheets.

**Figure 5:**
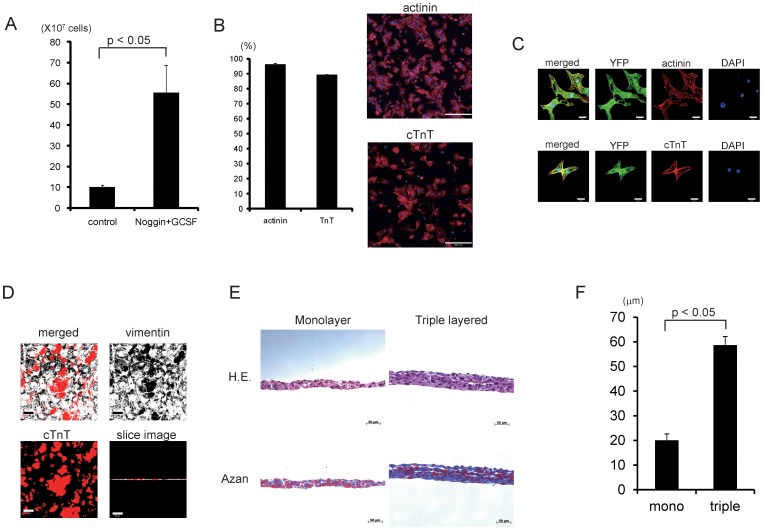
Liter-scale cultivation and cardiac cell sheet creation. (A) The number of remaining cells at 8 days after neomycin treatment (*n* = 4). (B) Almost all remaining cells after neomycin treatment were cardiomyocytes. Left, the percentage of contractive protein-expressing cells. Right, representative images of high content confocal microscopy to analyze cardiomyocyte purity. Cardiac contractive proteins (sarcomeric α-actinin, upper; cTnT, lower) are shown in red. Nuclei were counterstained with DAPI (blue). Bars, 200 µm. (C) Representative images of cardiac contractive protein expression (red) in the remaining cells after G418 selection in the bioreactor system. Nuclei were counterstained with DAPI (blue). Scale bars, 20 µm. (D) Distribution of cardiomyocytes (cTnT, red) and fibroblasts (vimentin, white) in a monolayer cardiac cell sheet. Scale bars, 50 µm. (E) Histological analyses of layered cardiac cell sheets. Stacked cell sheets were subjected to HE and azan staining. Left panels are images of monolayered sheets. Right panels are images of triple-layered cell sheets. Scale bars, 200 µm. (F) Thicknesses of cell sheets (*n* = 3).

Because the 100-ml bioreactor might not have been large enough to generate a sufficient number of cardiomyocytes to create bioengineered cardiac tissue, we used a 1-L bioreactor with the continuous medium exchange system (Figure 1E) and R1 mES cells. EBs from two vessels (125 ml each) at day 3 were re-seeded into a 1-L bioreactor. From day 10 until day 18, cells were treated with G418. At day 18, the number of cells that remained was about 1×10^8^ (Figure 5B). Consistent with our previous reports showing that serial treatments with noggin and granulocyte colony-stimulating factor (GCSF) in mES cell suspension culture increased the number of cardiomyocytes, treatments with noggin and GCSF in the bioreactor system (Figure 1E) significantly increased the number of remaining cells to around 5.5×10^8^ (Figure 5B). Almost all remaining cells showed spontaneous beating (Video S3), and high-content screening confocal microscopic analysis revealed that about 98% and 86% of the remaining cells were positive for sarcomericα-actinin and cardiac troponin T, respectively (Figure 5B, C). These findings suggest that the liter-scale bioreactor system with the continuous medium exchange system might enable generation of a large number of cardiomyocytes.

Finally, we attempted to fabricate cardiac tissue by layering cardiac cell sheets. Based on our previous reports [16], co-culture of mES cell-derived cardiomyocytes with fibroblasts at ratios of 8∶2 enables the creation of cardiac cell sheets (Figure 5D, E). Immunocytochemical analysis revealed that mES cell-derived cardiomyocytes in cell sheets were surrounded by vimentin-positive cells (Figure 5D). Moreover, using gelatin stamp methods [17], mES cell-derived cardiac cell sheets were layered in up to three layers. The maximal thickness of mES cell-derived cardiac sheets was approximately 20 µm for monolayer cell sheets and about 60 µm for triple-layered cell sheets (Figure 5E, F). Furthermore, even after triple layering, the cardiomyocytes in cell sheets showed vigorous spontaneous beating (Video S4), suggesting that layered mES-derived cardiac cell sheets have the potential to form cardiac tissue.

## Discussion

Various types of tissue engineering methods including cell sheets have been applied for creating heart tissue [18]. We previously reported creating the cardiac tissue by layering cardiac cell sheets derived from neonatal rat cardiomyocytes. However, mammalian cardiomyocytes lose their proliferative ability soon after birth [10]; thus, pluripotent stem cells such as ES cells and iPS cells are promising cell sources of cardiomyocytes. Recent reports have suggested that the transplantation of several layered cardiac cell sheets derived from mouse and human ES/iPS cells improves the cardiac function after myocardial infarction, however the effects are mediated through mainly paracrine fashion and almost all of transplanted cells are undetectable in 1–2 months [7], [19]. Therefore it might be a prerequisite to create transplantable bioengineered thickened cardiac tissue that directly contributes to contraction. When cardiac cell sheets are layered without vascularization in vivo, the three-layer construct is limited by thickness, possibly due to hypoxia, nutrient insufficiency and waste accumulation. On the other hand when triple layered pre-vascularized cardiac cell sheets from neonatal rats were transplanted onto the infarcted heart, many transplanted cell survived and contributed to the improvement of cardiac function [3], [20]. Furthermore we also previously developed 1-mm-thick myocardial tissue (total 30 layered) by the repeated layering of pre-vascularized triple layered neonatal rat cardiac cell sheets [9]. Although the development of the methods to vascularize the tissue using the in vitro vascular bed might be the big challenge for fabricating the thickened tissue, it also remains unclear whether ES-derived cardiomyocytes produced by the bioreactor system are suitable for collecting enough amounts of viable cardiomyocytes to create the functional pulsatile cardiac cell sheets.

In the present study, we have shown the utility of a stirred bioreactor culture system for generating large numbers of cardiomyocytes to create cardiac cell sheets. Recently, we reported that suspension culture using a spinner flask (250 ml) is useful for generating 4.9×10^6^ cardiomyocytes [16]. However, because culture conditions, such as pH and dissolved oxygen, cannot be regulated in a spinner flask, this approach might not be suitable for high-density culture. In this study, we used a bioreactor system equipped with a pH, dissolved oxygen and temperature regulatory system. When R1 ES cells were cultured in the 100-ml bioreactor with intermittent medium exchange, the number of purified cardiomyocytes was 1.5×10^7^ at day 18, twice the number of cells obtained from the same vessels without regulation (Figure 4D). Thus, regulation of cell culture condition might promote cardiac differentiation of mES cells.

High-density cultures consume a lot of nutrients, including glucose, and produce wastes such as lactate, which might lead to affect the cell proliferation and differentiation. The bioreactor culture with the continuous medium exchange system prevented lactate accumulation, the marked decrease in pH and promoted cell proliferated until at least day 10. The cell density and percentage of cardiomyocytes at day 10 were, respectively, 1.7-fold (Figure 3A) and 1.6-fold (Figure 4B) greater than those obtained in the bioreactor with the intermittent medium exchange system. The enhancement of cardiomyocyte generation in the continuous medium exchange was also confirmed by the results after G418 treatment (Figure 4D). Consequently, the liter scale bioreactor culture in a single run without growth factors enabled to produce cardiomyocytes enough to fabricate over 40 cardiac cell sheets derived from 3.5cm temperature responsive culture dishes.

The quality of cardiomyocytes is another important aspect in the culture methods for collecting cardiomyocytes, because their viability directly affects the cell sheets functional pulsation that is one of the indispensable properties of the cardiac tissue. Furthermore recent report has suggested that cardiomyocytes in the cell sheet mainly secrete the angiogenic growth factor when in transplantation, which leads to the ameliorate the ischemia-mediated cardiac injury and consequently improve the cardiac function [7]. Collectively the viability of cardiomyocytes after the differentiation process might be important from the view point of the contractile elements and the paracrine effects. Even though the same number of cardiomyocytes was seeded with same number of fibroblasts onto culture dishes, cardiomyocytes cultured with continuous medium exchange showed better engraftment in cell sheets in vitro compared with control cardiomyocytes or those cultured with intermittent medium exchange. These findings suggest that continuous optimization, but not intermittent optimization, of cell culture conditions might be critical for not only proliferation and differentiation of cardiomyocytes in a high-density cell culture, but also survival in cell sheets, which might lead to be capable of fabricating the sufficient number of functional cardiac cell sheets. There is currently little direct evidence to clarify the relationship between cardiomyocyte differentiation and physiological culture conditions. However, several reports have suggested that acidosis promotes hypoxia-mediated apoptosis in cardiomyocytes [21], [22] and sublethal hypoxia has been reported to induce ventricular dilation and myocardial hypoplasia in the fetal heart [23]. Thus, minimizing acidosis with a continuous medium exchange system might prevent cardiomyocyte death while promoting cardiomyocyte differentiation and proliferation. This would then lead to the generation of a large number of cardiomyocytes and better engraftments.

The three dimensional suspension culture methods have been wildly used for the efficient growth and differentiation of ES cells with microcarriers [24], [25] or EB formation [11], [13]. Microcarriers provide an enlarged attachment surface in a relatively small reactor volume due to their high surface area to volume ratio, however the necessity to use the animal-derived components for prior-coating might make the culture methods with microcarriers difficult to apply for the future clinical-grade cell preparations [26]. On the other hand, EB suspension culture is attractive in terms of the easily scalability and not obviating the need for removal of cells from microcarriers. Schroeder et al reported the development of a robust and scalable bioprocess that allows direct EB formation in a fully controlled, stirred bioreactor. They cultured mES cells with 8-bladed pitched turbine at 65 rpm and in 40% saturated O2, and aeration was performed through sparging with Antifoam C. Using this system, 1.28×10^9^ cardiomyocytes were generated from 2.0×10^8^ mES cells in a single 2-L bioreactor run with retinoic acid treatment [27]. Furthermore, the same group showed that a bioreactor with automated perfusion enabled the generation of 4.6×10^9^ cardiomyocytes from 2.0×10^8^ mES cells in a single 2-L bioreactor run [13]. Although the bioprocess for collecting robust cardiomyocytes have been already reported, there have not been studies to clarify the benefits of the bioreactor culture in terms of viability of cardiomyocytes to apply for the tissue engineering. In this study, we showed the evidences on the benefits of the bioreactor culture for the first time in the view points of not only the cardiomyocytes number, but also the engraftment of cardiomyocytes in the cell sheets.

We previously reported that the three-layer construct of cardiac cells sheets is limited by thickness, possibly due to hypoxia, nutrient insufficiency and waste accumulation. Consistent with this evidence, the thickness of three-layered mES cell-derived cardiac cell sheets was approximately three-fold that of monolayered cardiac cell sheets and the tissue did not contain any necrotic areas (Figure 5E). We also previously developed 1-mm-thick myocardial tissue by layering pre-vascularized cardiac cell sheets [9].Although cardiac cell sheets derived from mES cells did not contain vascular cells such as endothelial cells in the present study,the development of cardiac cell sheets with mES cell-derived endothelial cells is now ongoing. The bioreactor in the present study might generate cardiomyocytes that are healthier and more suitable for engraftment and for creating more functional cardiac tissue in a large scale, though we did not examine the definite benefits of the fabricated cardiac cell sheets for the field of heart repair. The functional benefits of thickened and vascularized cardiac tissue for regenerative medicine and disease models will be evaluated in the near future.

## Materials and Methods

### Animals and Reagents

Wild-type C57BL/6 mice were purchased from Japan SLC (Shizuoka, Japan). All protocols were approved by the Institutional Animal Care and Use Committee of Tokyo Women’s Medical University (Approval ID: 11-56-3-B3, 12-06-2). The following antibodies were used for immunocytochemistry: anti-sarcomeric α-actinin (Sigma-Aldrich, St. Louis, MO), anti-cardiac troponin T and anti-myosin heavy chain (Thermo Scientific, Rockford, IL) mouse monoclonal antibodies and a guinea pig monoclonal anti-vimentin antibody (PROGEN, Heidelberg, Germany). Secondary antibodies were purchased from Jackson ImmunoResearch Laboratories (West Grove, PA). Unless specified otherwise, reagents were purchased from Sigma-Aldrich.

### Mouse ES Cell Culture

Mouse ES cells (EMG7) expressing EGFP under the control of the α-myosin heavy chain promoter were maintained as described elsewhere [28]. R1 ES cells ubiquitously expressing EYFP, the neomycin phosphotransferase gene under the control of the α-myosin heavy chain promoter and phosphoglycerate kinase upstream of the hygromycin-resistance gene, were maintained as described elsewhere [29].

### Bioreactor System

Schemes of the bioreactor culture systems are shown in Figure 1A. A two-bladed (100-ml culture vessel) or three-bladed (1-L culture vessel) pitched turbine was used. The bioreactor (Bio Jr. 8 for 100 ml culture, BCP for 1 L culture; ABLE Co. Tokyo, Japan) was equipped with a temperature sensor (STE-03S180, ABLE), pH (EASYFERM PLUS 120, Hamilton, NV) and dissolved oxygen (InPro6800/12/120, Mettler Toledo, Columbus, OH) electrodes, as well as inoculation, harvest and sample ports. Data acquisition and process control were performed using a digital control unit and process control software (ABLE) for the MiniJar8 100-ml bioreactor and for the liter-scale bioreactor. Head-space aeration was performed. Dissolved oxygen was maintained at 40% with air, oxygen or nitrogen. The pH was maintained at 7.2 by CO_2_ addition when the culture medium was alkaline; when the medium was acidic, the pH was not regulated. The temperature was maintained at 37°C. The flow of EBs in the vessel was recorded using a Motion Analyzing Microscope (Keyence, Tokyo, Japan) with a CCD camera (VW-100C; Keyence). In some experiments, vessels equipped with sensors that did not regulate conditions were used in the CO2 incubator as a control.

### Bioreactor Culture

For the experiments to examine cardiac differentiation efficacy (Figure 1D), we cultured EMG7 ES cells using a 100-ml culture vessel with Glasgow’s Minimum Essential Medium (Invitrogen) supplemented with 10% FBS, nonessential amino acids (Invitrogen) and sodium pyruvate. Following trypsinization, 1×10^7^ mES cells were re-suspended in 100 ml of differentiation medium (1×10^5^ cells/ml), and seeded into a 100-ml stirred bioreactor (Bio Jr. 8; ABLE). On day 3, EBs cultured in a vessel were collected and re-seeded into four new vessels under the same conditions, and the culture medium was completely changed every day (100 ml/day) or continuously exchanged (100 ml/day).

For the collection of cardiomyocytes to create cell sheets (Figure 1E), 1×10^7^ R1 mES cells were cultured using 100-ml or 1-L culture vessels with DMEM supplemented with 15% FBS, nonessential amino acids, sodium pyruvate and L-glutamine (Invitrogen). For the 100-ml culture, EBs in a vessel at day 3 were re-seeded into four new vessels under the same conditions, and the culture medium was completely changed every day (100 ml/day) or continuously exchanged (100 ml/day). For the 1-L culture, EBs in two vessels at day 3 were re-seeded into a 1-L vessel under the same condition except for the agitation rate (100-ml culture, 85 rpm; 1-L culture, 65 rpm). The culture medium was continuously exchanged (1 L/day). For the 1-L culture with growth factors, the cells were cultured with noggin (150 ng/mL) from 3 days before to 1 day after the start of bioreactor culture and were cultured with GCSF (1 ng/mL) from day 6 to day 10 of the bioreactor culture. The cell number at each time point was measured after EB dissociation with 0.25% trypsin/EDTA. For cardiomyocyte purification, 400 µg/ml G418 was added to cultures from day 10 of cardiac differentiation until day 18. In some experiments, R1 ES cells were cultured in a similar 100-ml bioreactor vessel without regulation in the CO2 incubator as a control.

### Flow Cytometric Analysis and Fluorescence-activated Cell Sorting

EBs at each time point were dissociated with 0.25% trypsin/EDTA for 30 min, and the percentage of GFP(+) cells was analyzed using a Gallios (Beckman Coulter, Brea, CA) and Cell Quest Pro version 5.2 software. For cell sorting, a MoFlo XDP (Beckman Coulter) was used according to the manufacturer’s instructions. EB5 mES cells were used as a negative control.

### Biochemical Analysis

The culture supernatant was collected from the bioreactor culture system daily. Glucose, lactate, glutamine and glutamate were measured using a biosensor (BF5; ABLE) according to the manufacturer’s instructions.

### RNA Extraction and RT-PCR

Total RNA extraction and RT-PCR were performed as described elsewhere [6]. Quantitative PCR was performed by an ABI 7500 real-time PCR system (Applied Biosystems, Foster City, CA) with a QuantiTect SYBR Green PCR Master Mix (QIAGEN, CA). PCR conditions were initial denaturation at 94°C for 15 sec, followed by 60 cycles of 55°C for 30 s and 72°C for 35 s, and then melting curve analysis at 60–95°C. The relative mRNA expression level was calculated using a standard curve of GAPDH mRNA levels. All samples were independently analyzed at least three times for each gene. Primer sequences were as follow; 5′-CTGAGGGCCAGGCAGGAGCACGAG-3′, and 5′-CTGTAGGGAGGGCTTCGGGCACTT-3′ for Oct3/4, 5′-ATGCCAAAGAAAGAAACGAC-3′, and 5′-AGAGGCTGTAGAACATGATT-3′ for Brachyury T, 5′-TCTGTGGTTCTGCGTGGAGA-3′ and 5′-GTATCATTTCCAACCACCCT-3′ for Flk-1, 5′-CAGTGGAGCTGGACAAAGCG-3′ and 5′-TAGCGACGGTTCTGGAATTT- for Nkx2.5, 5′- TCTCACTATGGGCACAGCAG 3′ and 5′- GGGACAGCTTCAGAGCAGAC-3′ for GATA4, 5′- TTCTCATGACCCAGGCAGAC-3′ and 5′- CGTGGGTGATGATGTAGCAG-3′ for MLC-2a, 5′-TGACCACACAAGCAGAGAGG-3′ and 5′- CCGTGGGTAATGATGTGGAC-3′ for MLC2v and 5′-TTCACCACCATGGAGAAGGC-3′ and 5′-GGCATGGACTGTGGTCATGA-3′ for GAPDH.

### Immunocytochemistry

Cells were fixed with 4% paraformaldehyde, and the immunostaining methods have been described elsewhere [6]. Samples were imaged by laser confocal microscopy (Carl Zeiss, Oberkochen, Germany; Molecular Devices, Sunnyvale, CA), fluorescence microscopy (Nikon, Tokyo, Japan) with NIS-Elements software (Nikon), and Image Express (Molecular Devices) with MetaXpress and AcuityXpress software (Molecular Devices).

### Cell Isolation

Cardiac fibroblasts were obtained from the hearts of neonatal mice (1–2 days of age) as described previously [30]. Cardiac fibroblasts from passage 4 were used in experiments. Immunocytochemical analyses revealed that >99% of the cardiac fibroblasts expressed vimentin, and did not express von Willebrand factor (endothelial cell marker; data not shown) or NG2 (pericyte marker; data not shown).

### Cell Sheet Preparation

Prior to seeding cells, the surface of temperature-responsive dishes (Upcell; CellSeed, Tokyo, Japan) was coated with FBS for 2 hours. A mixed cell suspension of ES cell-derived cardiomyocytes and dermal fibroblasts isolated from adult mice was plated onto the Upcell at 3.2×10^5^ cells/cm^2^, and cells were cultured in DMEM supplemented with 15% FBS at 37°C. After 2 days in culture, cell sheets were harvested and layered using the cell sheet stacking manipulation technique described elsewhere [17].

### Histological and Immunohistochemical Analyses of Cell Sheets

To observe cross-sections of multi-layered cell sheets, multi-layered cell sheets were fixed with 4% paraformaldehyde and routinely processed into 3 mm thick paraffin-embedded sections. Hematoxylin and eosin (HE) staining and azan staining were performed by conventional methods.

### Statistical Analysis

Data are presented as the means ± standard deviation. Differences between groups were evaluated by the Student’s *t* test or analysis of variance followed by Bonferroni’s correction to compare means. A value of *p*<0.05 was considered statically significant.

## Supporting Information

Figure S1 **The effects of dissolved oxygen concentration in the culture medium in the bioreactor system on the cell proliferation and cardiac differentiation.** Mouse ES cells were cultured in the bioreactor system with various dissolved oxygen concentrations. (A) The number of EBs, (B) cell concentration, (C) the percentage of EBs that contains GFP(+) cells and (D) the percentage of spontaneous beating EBs at day10 (*n* = 3). Data are mean ± s.d.
(TIFF)

Figure S2 **The effects of agitation rate of impeller in the bioreactor system on the cell proliferation.** Mouse ES cells were cultured in the bioreactor system with various agitation rate of impeller. (A) the number of EBs in a vessel and (B) cell concentration at day6 (*n* = 2). Data are mean ± s.d.
(TIFF)

Figure S3 **The comparison of mRNA expression levels of cardiac genes.** RNA was extracted at day 9 in in the bioreactor system with the intermittent or continuous medium exchange system (*n* = 3). Data are mean ± s.d.
(TIFF)

Video S1 **The agitation of EBs in the bioreactor.**
(WMV)

Video S2 **The spontaneous beating of cardiomyocytes in EBs cultured on a cell culture dish.**
(WMV)

Video S3 **The spontaneous beating of purified cardiomyocytes after the treatment with G418.**
(WMV)

Video S4 **The spontaneous beating of triple layered cardiac cell sheets.**
(WMV)
