# Supplementary figures and images for: Fabrication of Mouse Embryonic Stem Cell-Derived Layered Cardiac Cell Sheets Using a Bioreactor Culture System
Source: PLoS One. 2012 Dec 20;7(12):e52176. doi: 10.1371/journal.pone.0052176 (PMC3527435; doi:10.1371/journal.pone.0052176)

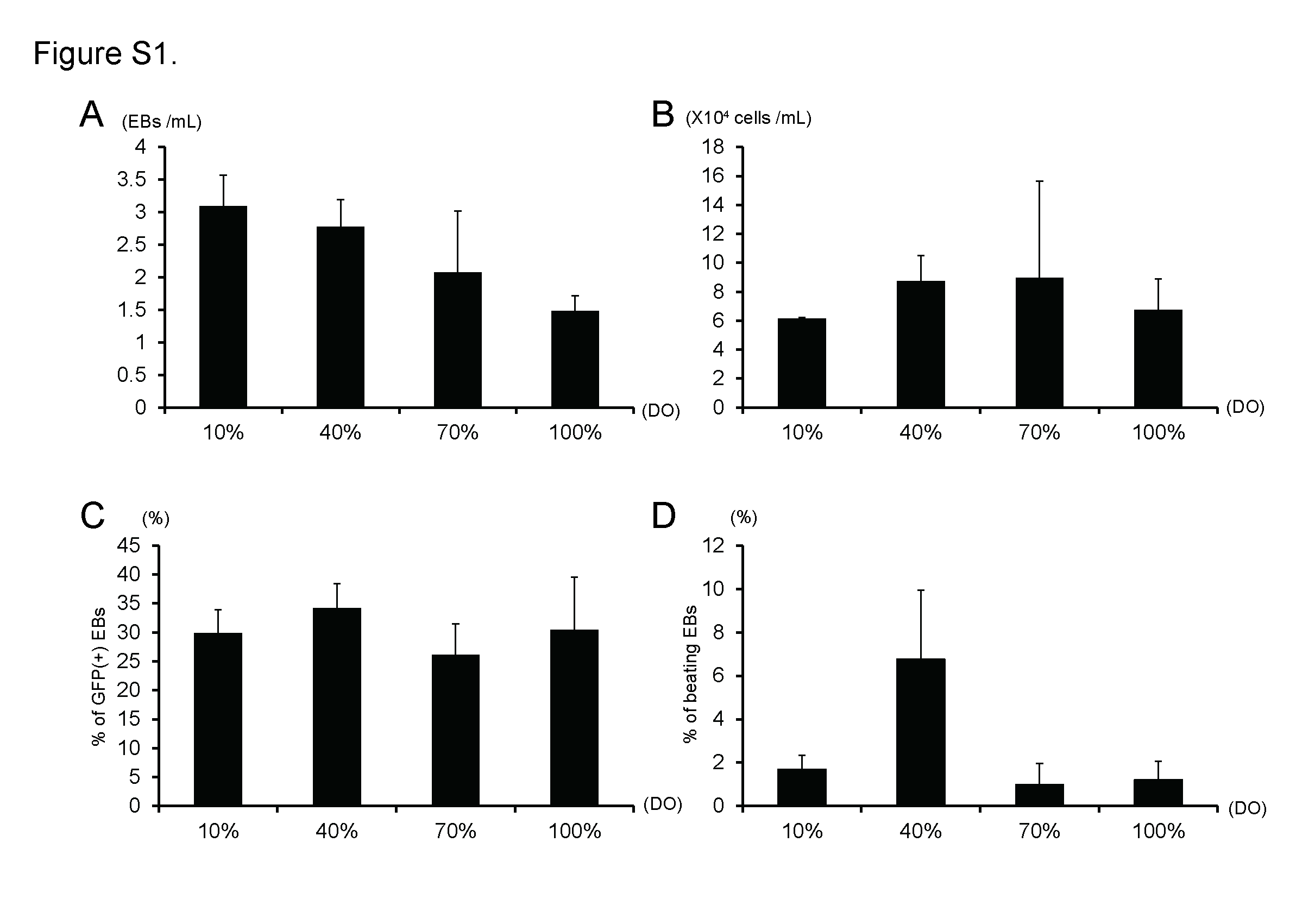

Supplement: Figure S1 — The effects of dissolved oxygen concentration in the culture medium in the bioreactor system on the cell proliferation and cardiac differentiation. Mouse ES cells were cultured in the bioreactor system with various dissolved oxygen concentrations. (A) The number of EBs, (B) cell concentration, (C) the percentage of EBs that contains GFP(+) cells and (D) the percentage of spontaneous beating EBs at day10 (n = 3). Data are mean ± s.d. (TIFF) [file pone.0052176.s001.tiff]

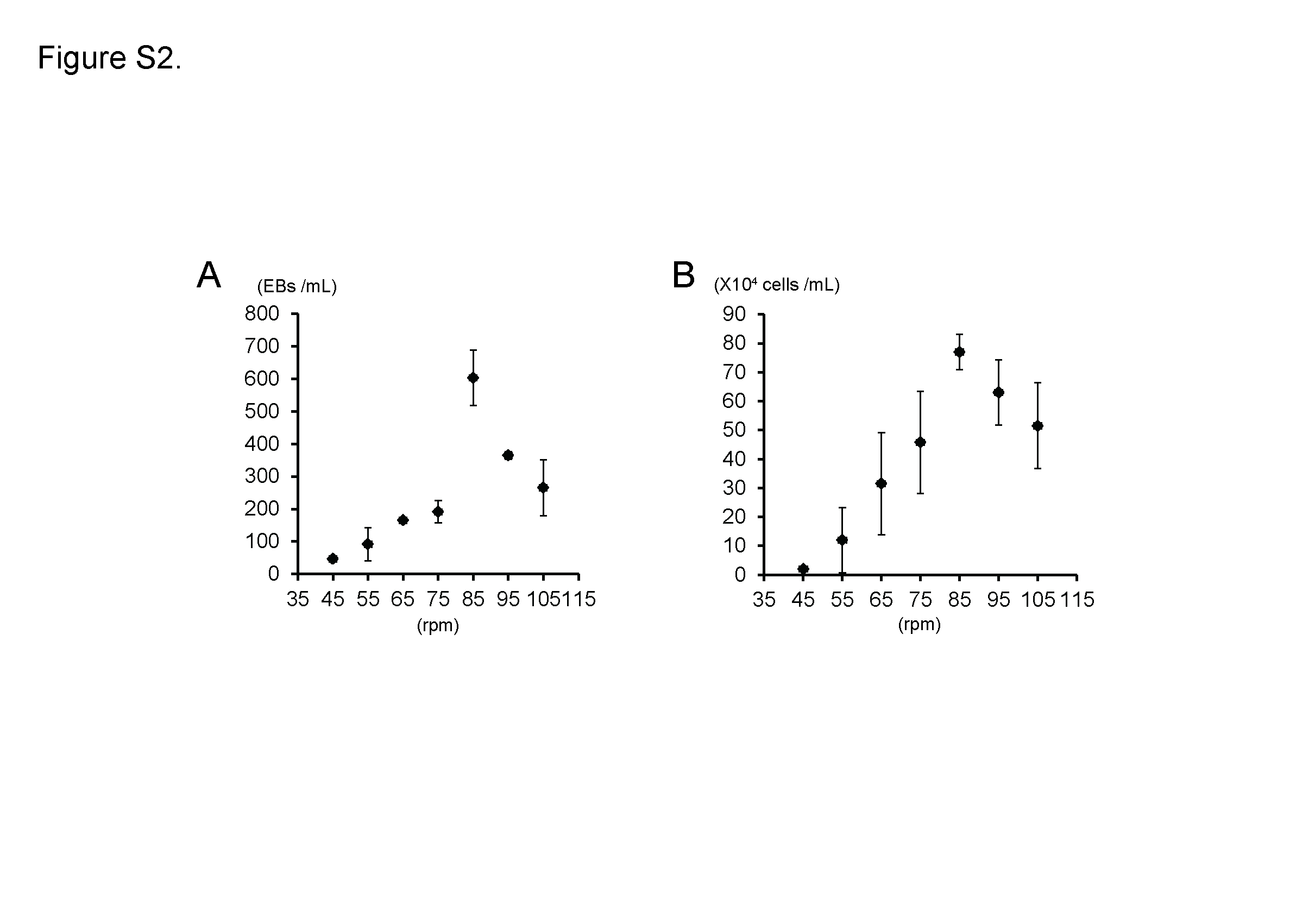

Supplement: Figure S2 — The effects of agitation rate of impeller in the bioreactor system on the cell proliferation. Mouse ES cells were cultured in the bioreactor system with various agitation rate of impeller. (A) the number of EBs in a vessel and (B) cell concentration at day6 (n = 2). Data are mean ± s.d. (TIFF) [file pone.0052176.s002.tiff]

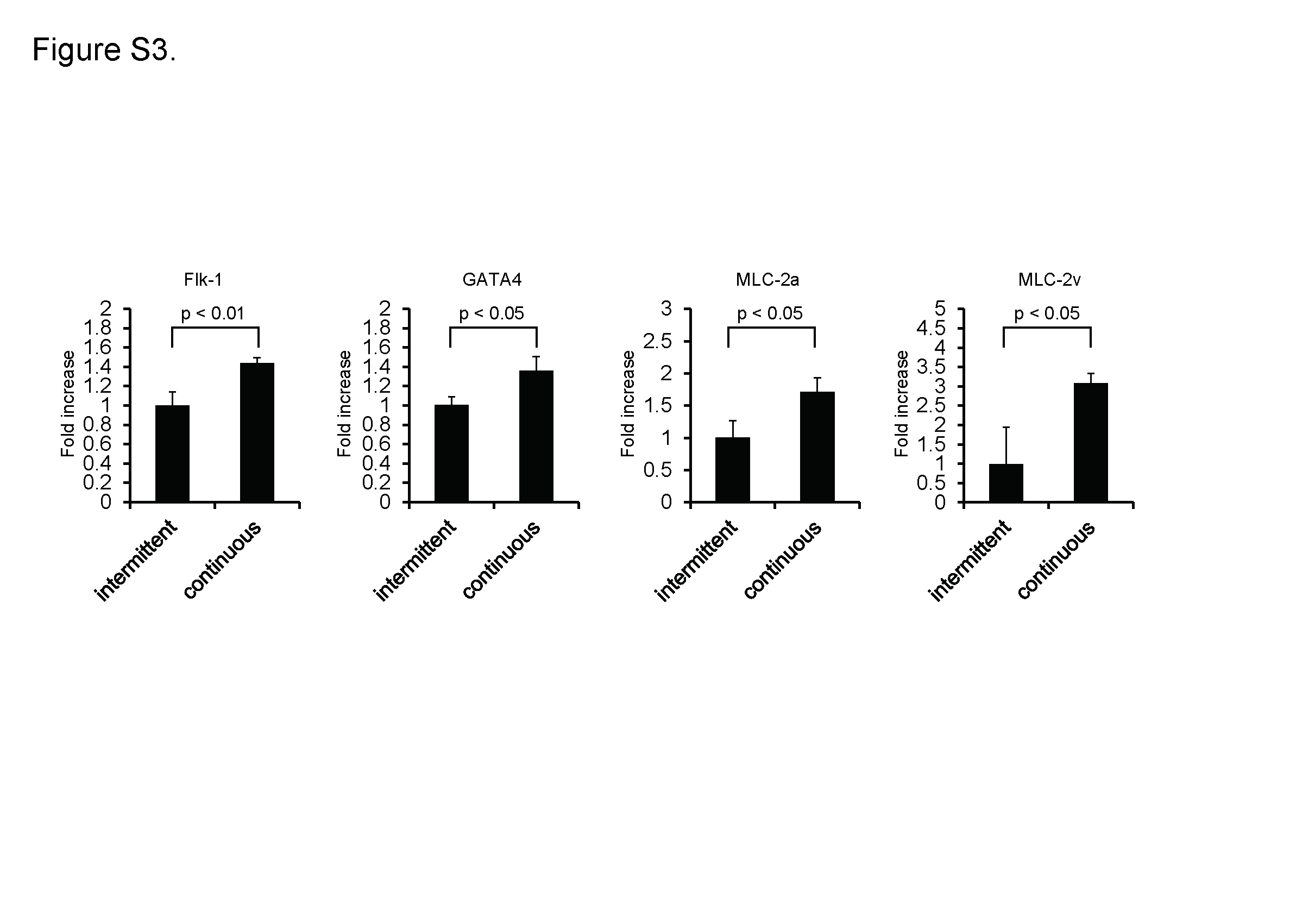

Supplement: Figure S3 — The comparison of mRNA expression levels of cardiac genes. RNA was extracted at day 9 in in the bioreactor system with the intermittent or continuous medium exchange system (n = 3). Data are mean ± s.d. (TIFF) [file pone.0052176.s003.tiff]
